# Effective Plug-Ins for Reducing Inference-Latency of Spiking Convolutional Neural Networks During Inference Phase

**DOI:** 10.3389/fncom.2021.697469

**Published:** 2021-10-18

**Authors:** Xuan Chen, Xiaopeng Yuan, Gaoming Fu, Yuanyong Luo, Tao Yue, Feng Yan, Yuxuan Wang, Hongbing Pan

**Affiliations:** The School of Electronic Science and Engineering, Nanjing University, Nanjing, China

**Keywords:** artificial neural network, spiking neural network, deep learning, object classification, deep networks, spiking network conversion, inference-latency

## Abstract

Convolutional Neural Networks (CNNs) are effective and mature in the field of classification, while Spiking Neural Networks (SNNs) are energy-saving for their sparsity of data flow and event-driven working mechanism. Previous work demonstrated that CNNs can be converted into equivalent Spiking Convolutional Neural Networks (SCNNs) without obvious accuracy loss, including different functional layers such as Convolutional (Conv), Fully Connected (FC), Avg-pooling, Max-pooling, and Batch-Normalization (BN) layers. To reduce inference-latency, existing researches mainly concentrated on the normalization of weights to increase the firing rate of neurons. There are also some approaches during training phase or altering the network architecture. However, little attention has been paid on the end of inference phase. From this new perspective, this paper presents 4 stopping criterions as low-cost plug-ins to reduce the inference-latency of SCNNs. The proposed methods are validated using MATLAB and PyTorch platforms with Spiking-AlexNet for CIFAR-10 dataset and Spiking-LeNet-5 for MNIST dataset. Simulation results reveal that, compared to the state-of-the-art methods, the proposed method can shorten the average inference-latency of Spiking-AlexNet from 892 to 267 time steps (almost 3.34 times faster) with the accuracy decline from 87.95 to 87.72%. With our methods, 4 types of Spiking-LeNet-5 only need 24–70 time steps per image with the accuracy decline not more than 0.1%, while models without our methods require 52–138 time steps, almost 1.92 to 3.21 times slower than us.

## 1. Introduction

CNN architectures, such as YOLO (Redmon et al., 2016), ResNet (He et al., 2016), GoogLeNet (Szegedy et al., 2015, 2016) and VGG-16 (Simonyan and Zisserman, 2014), have been successfully proved effective on computer vision benchmarks like ImageNet (Deng et al., 2009). Besides, there are many mature methods (e.g., forward propagation, back propagation, gradient descent; Rumelhart et al., 1986) and tools [e.g., PyTorch (Paszke et al., 2019), Caffe (Jia et al., 2014), Tensorflow (Abadi et al., 2016)] for training. However, higher accuracy of classification means larger and deeper CNN architectures, which further incurs more operands and larger energy costs. To overcome these challenges, many researchers made attempts to make their CNN architectures smaller or sparser by pruning (Guo et al., 2016; He et al., 2017), compression (Han et al., 2016) and quantization (Gong et al., 2014), displaying promising results. In spite of these methods, to achieve higher accuracy, deeper and more complicated neural networks still need a large amount of computing resources and power consumption (Tan and Le, 2019).

Imitating human's brain, SNN architectures replace particular data values in CNNs with spikes. The event-based operations in SNNs ensure low-power consumption in their hardware implementations, such as Field-Programmable Gate Arrays (FPGAs) (Han et al., 2020) and Application Specific Integrated Circuits (ASICs) (Frenkel et al., 2019). In addition, large-scale neuromorphic spiking platforms such as TrueNorth (Benjamin et al., 2014; Merolla et al., 2014) and SpiNNaker (Furber et al., 2014) are also useful for SNN simulation. Though SNNs are energy efficient, they are not as popular as CNNs on account of relatively immature training methods and worse accuracies (Roy et al., 2019). Training methods of SNNs include unsupervised spike-timing-dependent plasticity (STDP) (Diehl and Matthew, 2015) and supervised gradient descent and back-propagation (Haeng et al., 2016). Although STDP is biologically closer to human's brain, its learning performance is significantly lower than that of CNNs. Recent works (Jin et al., 2018) proposed a supervised learning algorithm, using an approximate function to represent the non-differentiable portion of SNNs in order to use back-propagation. Despite these efforts, most previous works can achieve good performance for MNIST dataset but still remain gaps for more difficult datasets (Roy et al., 2019).

Combining the effectiveness of CNNs with the efficiency of SNNs, Cao et al. (2015) raised SCNNs and described the way to directly transform CNNs to SNNs without complex changes. Specifically, spikes are generated by random number and probability. The frequency of spikes during a long time represents corresponding value in CNNs. Max-pooling layers are replaced by Avg-pooling layers. All the biases in Conv layers and FC layers are set to zero. This work reports good performance on CIFAR-10 dataset, error rates of which are not more than 2% less than that of original CNNs. Diehl et al. (2015) improved SCNNs by using a weight scheme, which rescaled the weights to avoid approximation errors in neurons. Rueckauer et al. (2017) changed the random input to constant analog activation, which improved accuracy and reduced inference-latency. In addition, their work extended SCNNs by developing spiking implementations of Max-pooling layers, softmax activation, neuron biases, and BN layers (Ioffe and Szegedy, 2015) in CNNs. Among large-scale CNNs, VGG-16 and GoogLeNet Inception3 have been successfully converted into spiking forms (Rueckauer et al., 2017). Spiking-ResNet and Spiking-YOLO have been implemented by later works (Sengupta et al., 2018; Kim et al., 2019).

Though these approaches have achieved good results on MNIST (Lecun et al., 1998), CIFAR-10 (Krizhevsky and Hinton, 2009) and ImageNet (Deng et al., 2009) datasets, during the simulation of Spiking-LeNet-5 and Spiking-AlexNet models, we find that the large inference-latency of SCNNs is an ineludible problem. Analysis of Rueckauer et al. (2017) is focusing on raising firing rates of neurons in each layer through weight-normalization, which will effectively save time. Zambrano and Bohte (2016) have developed a conversion method using spiking neurons with adaptive firing threshold to reduce the needed number of spikes for information encoding. Panda et al. (2016) added extra output layers to get part of results in advance. Neil et al. (2016) introduced several approaches during training phase to reduce inference-latency. Yang et al. (2020) proposed a novel n-scaling weight mapping method to realize high-accuracy and low-latency SCNNs. Though these works have achieved effective performance on reducing inference-latency of SCNNs, their focuses of attention are on weights, thresholds, network architectures and training phase, not on the end of inference phase.

Through observing output spike counts received by counters corresponding to neurons of the last layer in SCNNs, we find that there are some input data points hard to classify. To be specific, at least two maximal output spike counts always look similar. These input data points are defined as tough data. The number of tough data is decreasing with the increment of time steps. But even when time steps are large enough, there are still tough data remaining, leading to incorrect results. Compared with tough data, other data can obtain expected results and consume fewer time steps. Therefore, as long as we can distinguish tough data and other data as early as possible, inference-latency for other data can be greatly saved, reducing total inference-latency. During inference phase, according to real-time output spike counts, through analyzing the gap between output spike counts, we add different stopping criterions to determine whether current SCNN continues or generates result. These stopping criterions are based on observation, experiment results and mathematical demonstration. In cognitive neuroscience field, Sequential Sampling Models (Forstmann et al., 2015) and Visual Confidence (Mamassian, 2016) are models for organic brains to deal with determination problem. The connection between our stopping criterions and these models provides a biological basis for the reasonability of our methods.

In this work, we propose 4 stopping criterions to reduce the inference-latency of SCNNs during inference phase. As plug-ins at the end of networks, experiments demonstrate that our stopping criterions can significantly save total inference-latency without obvious accuracy loss. Compared with original models following existing techniques (Rueckauer et al., 2017), for Spiking-AlexNet and CIFAR-10, we only need 267 time steps per image to achieve the accuracy of 87.72%, the accelerative ratio of which is 3.34X. For Spiking-LeNet-5 and MNIST, we use 4 types of models, Avg-pooling with no biases, Avg-pooling with no biases but Poisson input, Max-pooling with no biases and Avg-pooling with BN layers and biases, respectively, verifying the universality and compatibility of our methods. For Spiking-LeNet-5 with Avg-pooling and no biases, 24 time steps per image can obtain the accuracy of 98.50% and the accelerative ratio is 2.3X. For Spiking-LeNet-5 with Avg-pooling and no biases but Poisson input, we can attain the accuracy of 98.48% with 27 time steps per image, while the accelerative ratio is 1.92X. For Spiking-LeNet-5 with Max-pooling and no biases, 30 time steps are needed per image to reach the accuracy of 97.91%. Its accelerative ratio is 3.21X. For Spiking-LeNet-5 with Avg-pooling and BN layers, to achieve the accuracy of 98.73%, 44 time steps are required, the accelerative ratio of which is 1.95X.

The remainder of this paper is organized as follows. Section 2 introduces the basic principle for CNNs converting to SCNNs, observations and analysis for training dataset and the 4 proposed stopping criterions to reduce inference-latency. Then, we give the software experimental results and compare our methods with the prior arts in section 3. In section 4, we discuss relative cognitive neuroscience works, compare the 4 stopping criterions and summarize our work.

## 2. Methods

In section 2.1, details of conversion from CNNs to SCNNs are provided, including information of CNNs, the implementation of Max-pooling layers and Batch-Normalization layers in SCNNs, neuron equations used for conversion, different input formats and rules for thresholds and weight normalization. In section 2.2, we observe original SCNN models described in section 2.1, analyze the relation between inference-latency and accuracy and find some data points difficult to obtain unique classification results along with time steps. Such data points are defined as tough data. In section 2.3, for training dataset, the relation between output spike counts in SCNNs and corresponding values in CNNs is analyzed. In section 2.4, we propose 4 stopping criterions to reduce the inference-latency for SCNNs.

### 2.1. Converting CNNs to SCNNs

The rules converting CNNs to SCNNs used in this paper are based on the work of Rueckauer et al. (2017), ensuring the universality and good performance of our original SCNN models.

#### 2.1.1. Network Architectures

In this work, we use AlexNet (Krizhevsky et al., 2012) for CIFAR-10 and LeNet-5 (Lecun et al., 1998) for MNIST. In the work of Cao et al. (2015), the conversion from CNNs to SCNNs requires replacing Max-pooling layers by Avg-pooling layers and setting biases to zero. Therefore, our main SCNN models use Avg-pooling layers and have no biases. In addition, we use LeNet-5 to test situations with Max-pooling layers and Batch-Normalization layers.

Rueckauer et al. (2017) created the way to implement Max-pooling layers in SCNNs according to the principle of Winner-Take-All. More specifically, in the Max-pooling window, only the earliest neuron generating spikes will be the choice. Besides, Rueckauer et al. (2017) successfully added biases and BN layers in their SCNNs. Through altering weights and biases of one Conv layer or FC layer, the following BN layer can be deleted. These new biases can be continuously added into corresponding neurons after dividing the product of thresholds before this layer.

The trained accuracy of AlexNet-avg-0b is 87.95%. The accuracy of LeNet-5-avg-0b after training is 98.56%. Then we replace Avg-pooling layers by Max-pooling layers. The accuracy of LeNet-5-max-0b is 98.65%. We also add two BN layers to LeNet-5. BN layer 1 follows Conv layer 1 and BN layer 2 follows Conv layer 2. All the layers in this model have biases. The accuracy of LeNet-5-avg-BN after training is 98.82%.

#### 2.1.2. Neuron

The basic principle of converting CNNs to SCNNs is that the frequency of spike in SCNNs is approximately linear to the value in CNNs. In neurons, spikes are generated by firing operations. According to the work of Rueckauer et al. (2017), the membrane potential V(t) of a spiking neuron in SCNN models is updated at each time step by the following equations derived from the integrate-and-fire neuron model:


(1) $$\begin{array}{l} \begin{array}{c} V\left(t\right)=V\left(t-1\right)+X\left(t\right)\\ if\;V\left(t\right)\geq V_{thr},\;spike\;and\;reset\;V\left(t\right)=V\left(t\right)-V_{thr}. \end{array} \end{array}$$

In the neuron equations, X(t) is the sum of all the input synapses connected into the neuron. Particularly, X(t) is the dot product of input vector and weight vector. Input vector comes from input activation or output spikes of other neurons. Weight vector means strength of synapses in neurons. The correspondence between input vector and weight vector is the same as that in CNNs. After V(t) exceeds its threshold V_*thr*_, the neuron fires and generates a spike, transmitted to neurons in the next layer. Meanwhile, the neuron's membrane potential V(t) will be reset to V(t)-V_*thr*_.

#### 2.1.3. Thresholds and Normalization

In the papers of Diehl et al. (2015) and Rueckauer et al. (2017), all the thresholds were set to 1, altering and normalizing weights and biases simultaneously. In our work, we use 99.9% normalization. For each layer and each input data point in training dataset, we calculate the dot product of input vector and weight vector in CNNs, named output vector. For network with biases, the output vector also needs to add the bias vector. Then we delete zero and negative values in output vectors and find the 0.1% maximal value from the rest for each layer. By pervious operations, we can get one value per layer, defined as ${\text{O}}_{layer}^{0.1\%}$. The weights ${\text{W}}_{layerN}^{SCNN}$ and biases ${\text{B}}_{layerN}^{SCNN}$ in layer N will be transformed by following equations:


(2) $$\begin{array}{l} \begin{array}{c} W_{layerN}^{SCNN}=W_{layerN}^{CNN}*\frac{O_{layerN-1}^{0.1\%}}{O_{layerN}^{0.1\%}}\\ B_{layerN}^{SCNN}=B_{layerN}^{CNN}*\frac{1}{O_{layerN}^{0.1\%}}\\ O_{layer0}^{0.1\%}=1. \end{array} \end{array}$$

#### 2.1.4. Input

In the paper of Rueckauer et al. (2017), input data for SCNNs are in the form of continuous analog currents between 0 and 1. For validation of universality, we also use Poisson spikes as our input for LeNet-5-avg-0b model. According to the work of Cao et al. (2015), we transform input data I in MNIST dataset ranging from 0 to 255 into Poisson spikes by following rules:


(3) $$\begin{array}{l} \begin{array}{c} randomnumber\left(t\right)\sim U\left(0,1\right)\\ if\;randomnumber\left(t\right)<I/255,I^{Poisson}\left(t\right)=1\\ if\;randomnumber\left(t\right)\geq I/255,I^{Poisson}\left(t\right)=0. \end{array} \end{array}$$

The random number is generated per time step t and follows uniform distribution pattern from 0 to 1. I^*Poisson*^(t) means the input spike transmitted into SCNNs at time step t.

### 2.2. Observation of Original SCNN Models

After all the steps introduced in section 2.1, we have built our original SCNN models. Observation of these models during inference phase is presented in this section.

#### 2.2.1. Original Stopping Criterion

For original models, all the data have been run for the same set finish time. For the last layer, one counter per neuron is used to count the number of output spikes. Then we compare all the counters after finish time, the maximal counter of which will be the winner. The set finish time is defined as T_*max*_. The unit of T_*max*_ is time step. We use original stopping criterion to describe this termination operation.

#### 2.2.2. Random Selection for Tough Data

During our observation, not matter how many time steps have been consumed, there still remain some input data points difficult to classify. For these input data points, at least two maximal counters always receive almost equivalent number of spikes. In this case, different T_*max*_ may lead to different results. We use tough data to represent such situations. On the contrary, maximal counter of other data can obviously receive more spikes than other counters.

In the code, the way to find maximal counter can directly determine the classification result of tough data. For example, ascending or descending order will lead to different accuracies, drawing imprecise conclusions. To avoid this problem, 10,000 times of random selections between the same maximal counters will be used during each determination of tough data. The final accuracy is an average result of 10,000 times.

#### 2.2.3. The Influence of T_*max*_ on Accuracy and Tough Data

**Figure 1:**
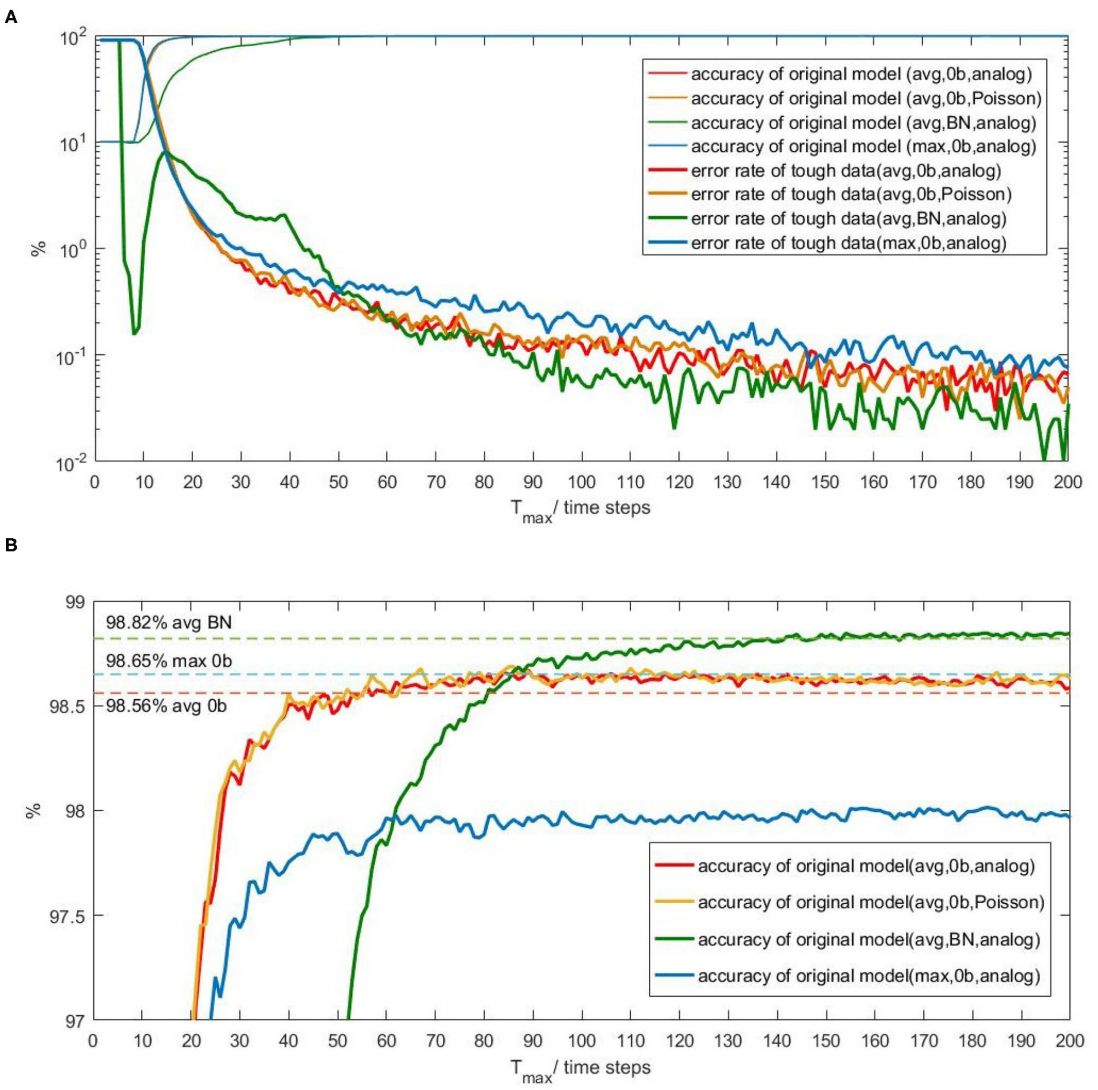
The relation between inference-latency and accuracy and the relation between inference-latency and error rate of tough data. **(A)** Four thick lines are error rates produced by tough data for 4 different Spiking-LeNet-5 models. Four fine lines are accuracies of these models. **(B)** The accuracy in the range of 97 to 99%. CNN accuracy for each model has been drawn by dotted lines.

Figure 1A shows that the accuracy of each Spiking-LeNet-5 original model is close to 10% at first several time steps and then increases significantly. After a sudden increasing phase, each accuracy increases slowly and tends to be stable. The accuracy of BN model increases much slower than that of other models. After zooming in on Y-axis, Figure 1B clearly shows that accuracies are rising slowly in fluctuation after the sudden increasing phase. Spiking-LeNet-5 (avg, 0b), no matter analog input or Poisson input, can easily achieve its CNN accuracy (98.56%) when T_*max*_ is larger than 60 time steps. Spiking-LeNet-5 (avg, BN) can also achieve its CNN accuracy (98.82%) but consumes more time steps than other models. The accuracy achieved by Spiking-LeNet-5 (max, 0b) is not more than 98%, lower than CNN accuracy (98.65%). This gap comes from the implementation of Max-pooling. T_*max*_ larger than 60 time steps guarantees an accuracy higher than 97.9%.

Besides, error rates contributed by tough data are presented in Figure 1A. For models with no biases, such error rates decrease dramatically at first and slowly later on. Error rate of Max-pooling model is higher than Avg-pooling models with no biases, in accord with accuracy results. For BN model, error rate generated by tough data first drops down and then rises up to normal level like other models. This phenomenon is caused by biases. At first several time steps, neurons with relatively large biases will generate spikes early, reducing the number of tough data. Then spikes mainly contributed by input will be generated. Output spike counts gradually move close to ideal results. From the perspective of time, we can infer from Figure 1A that classifying tough data needs long time, even not ensuring correct results. On the contrary, classifying other data needs shorter time. To reduce inference-latency, different strategies should be used for different types of data.

### 2.3. Output Spike Analysis

To better learn characteristics of tough data and other data, output spike counts should be analyzed carefully, together with their corresponding values in CNNs.

#### 2.3.1. The Relation Between Output Spike Counts and Corresponding Values in CNN

**Figure 2:**
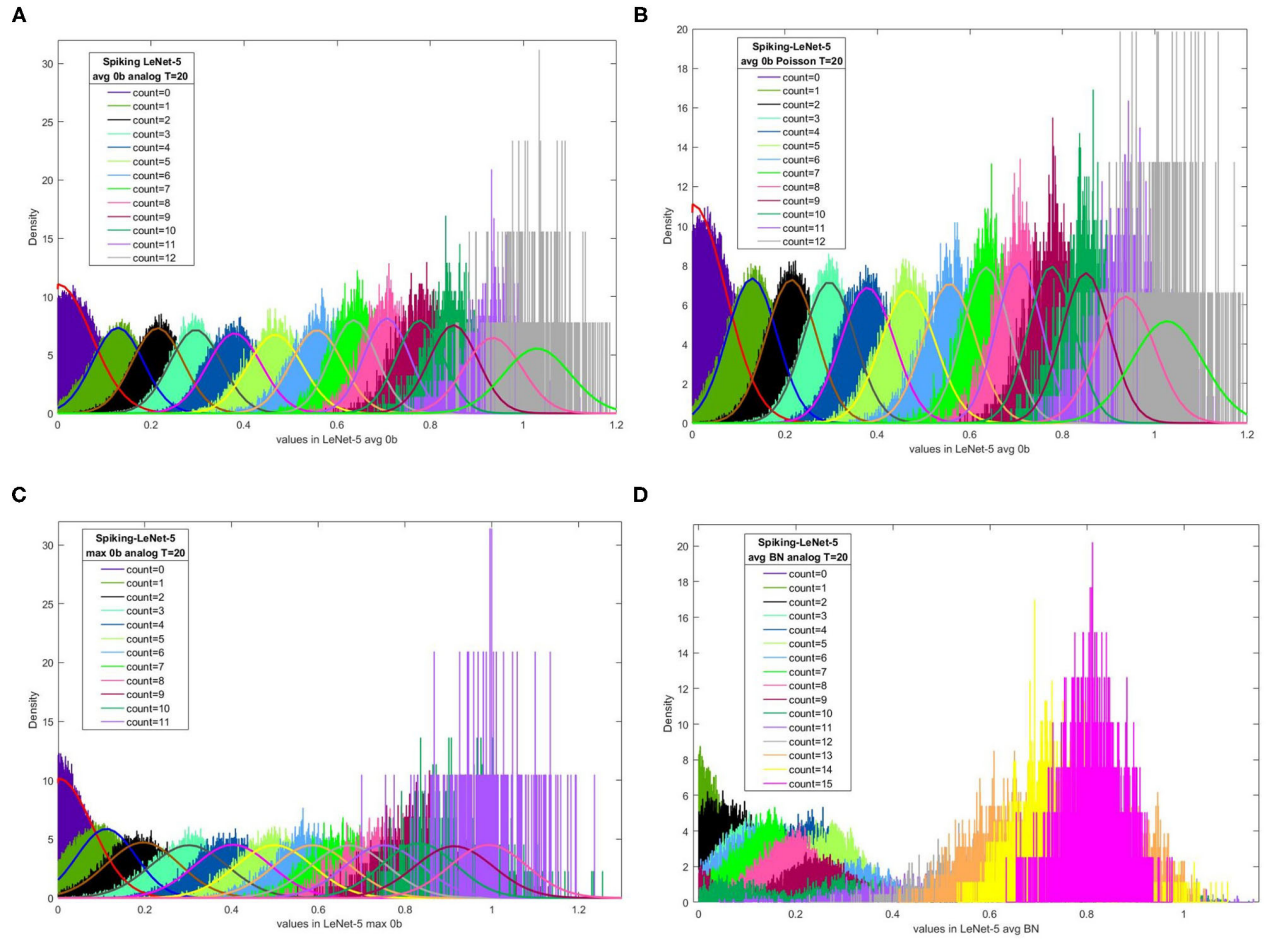
The relation between output spike counts in Spiking-LeNet-5 and corresponding CNN values distribution (greater than zero) in LeNet-5 after T time steps. Distributions with different colors correspond to different output spike counts. For models without biases, normal fitting or half-normal fitting are added. **(A)** Spiking-LeNet-5 avg 0b analog *T* = 20 time steps, **(B)** Spiking-LeNet-5 avg 0b Poisson *T* = 20 time steps, **(C)** Spiking-LeNet-5 max 0b analog T=20 time steps, **(D)** Spiking-LeNet-5 avg BN analog *T* = 20 time steps.

Figures 2A–D present the relation between output spike counts in Spiking-LeNet-5 and corresponding value distribution (greater than zero) in LeNet-5 after T time steps, for Spiking-LeNet-5 (avg, 0b, analog input) model, Spiking-LeNet-5 (avg, 0b, Poisson input) model, Spiking-LeNet-5 (max, 0b, analog input) model and Spiking-LeNet-5 (avg, BN, analog input) model, respectively. All the input data points are from MNIST training dataset (60,000 images). In these figures, X-axis means value in CNNs and Y-axis means density. Data in these figures colored diversely correspond to different output spike counts in SCNNs. For one fixed CNN value, several output spike counts can be generated by SCNN. For one fixed output spike count, the probability distribution of its corresponding CNN value is clearly shown in these figures. Obviously, for models with no biases, we can use normal fitting to estimate these probability distributions. For models with biases, unfortunately, these probability distributions look like a mess. We need to find other ways to analyze models with biases.

#### 2.3.2. Normal Fitting Parameters for Models With No Biases

As output spike count 0 corresponds to most of negative values and part of relatively small positive values in CNNs, we cannot use normal fitting to deal with this asymmetric situation. Besides, negative values are not important during final classifying phase. Only positive values need to be analyzed. In consequence, for positive values corresponding to output spike count 0, we use half-normal fitting with a mean zero to get the standard deviation.

**Figure 3:**
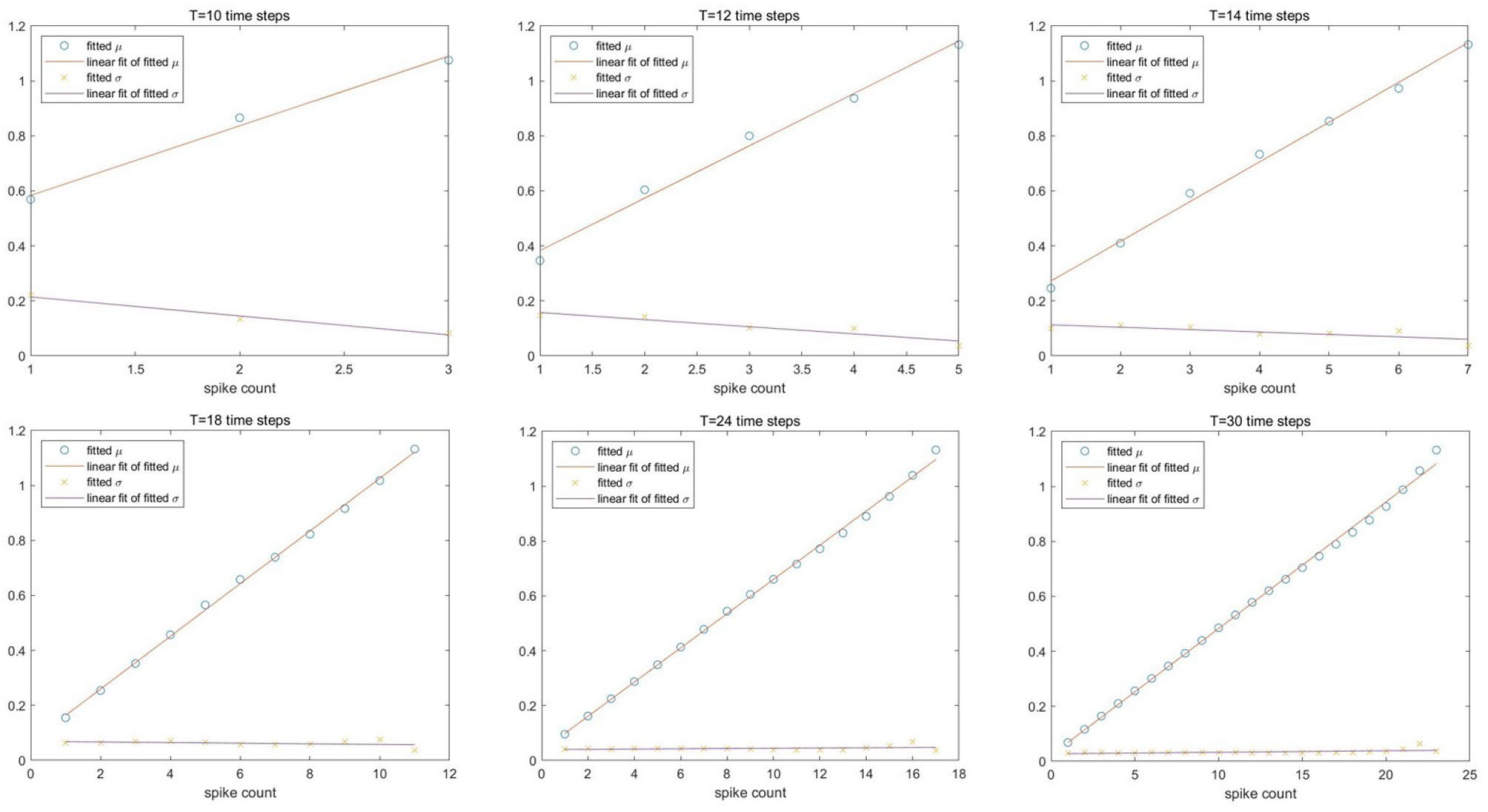
Linear fitting of normal fitting parameters after different time steps for Spiking-LeNet-5 avg 0b analog model. The upper lines are linear fitting of means and the other lines are linear fitting of standard deviations. X-axis is output spike count.

For Spiking-LeNet-5 (avg, 0b, analog input), Figure 3 presents its normal fitting parameters after different time steps. In each subfigure, the upper oblique line is the linear fitting result of fitted means and the other line is the linear fitting result of fitted standard deviations. X-axis means output spike counts. No matter how many time steps have been consumed, fitted means have a good linear relation with output spike counts, while fitted standard deviations are close to each other. Similar results can be obtained in Spiking-LeNet-5 (avg, 0b, Poisson input) and Spiking-LeNet-5 (max, 0b, analog input), but fitted standard deviations of Spiking-LeNet-5 (max, 0b, analog input) are obviously larger than those of Avg-pooling models. This difference probably comes from the implementation of Max-pooling.

#### 2.3.3. Focusing on Max1 and Max2 CNN Values for Models With Biases

According to Figure 2D, we cannot use normal fitting to describe the relation between output spike counts and CNN values for models with biases. We need to analyze from other points of view. From Figure 2D, we can see that relatively small CNN values (less than 0.4) may belong to several different output spike counts. For one fixed CNN value in this range, it's difficult for us to estimate its corresponding output spike count. Similarly, for two fixed output spike counts in this range, we are not certain that the CNN value corresponding to the larger output spike count is larger as well. Nevertheless, from Figure 2D we can also find that relatively large CNN values (greater than 0.4) belong to larger output spike counts. The distribution of CNN values for relatively small output spike counts is obviously separate from that for relatively large output spike counts. In this case, if we observe one CNN value less than 0.4 and another CNN value greater than 0.4, we will find the output spike count of larger CNN value larger with high probability. In CNNs, we always find maximal value among neurons in the last layer to get classification result. If we only take maximal CNN value and second maximal CNN value into consideration, distributions of relatively small values may be filtered out. Avoiding dealing with disordered distributions, this perspective can help us make decisions.

**Figure 4:**
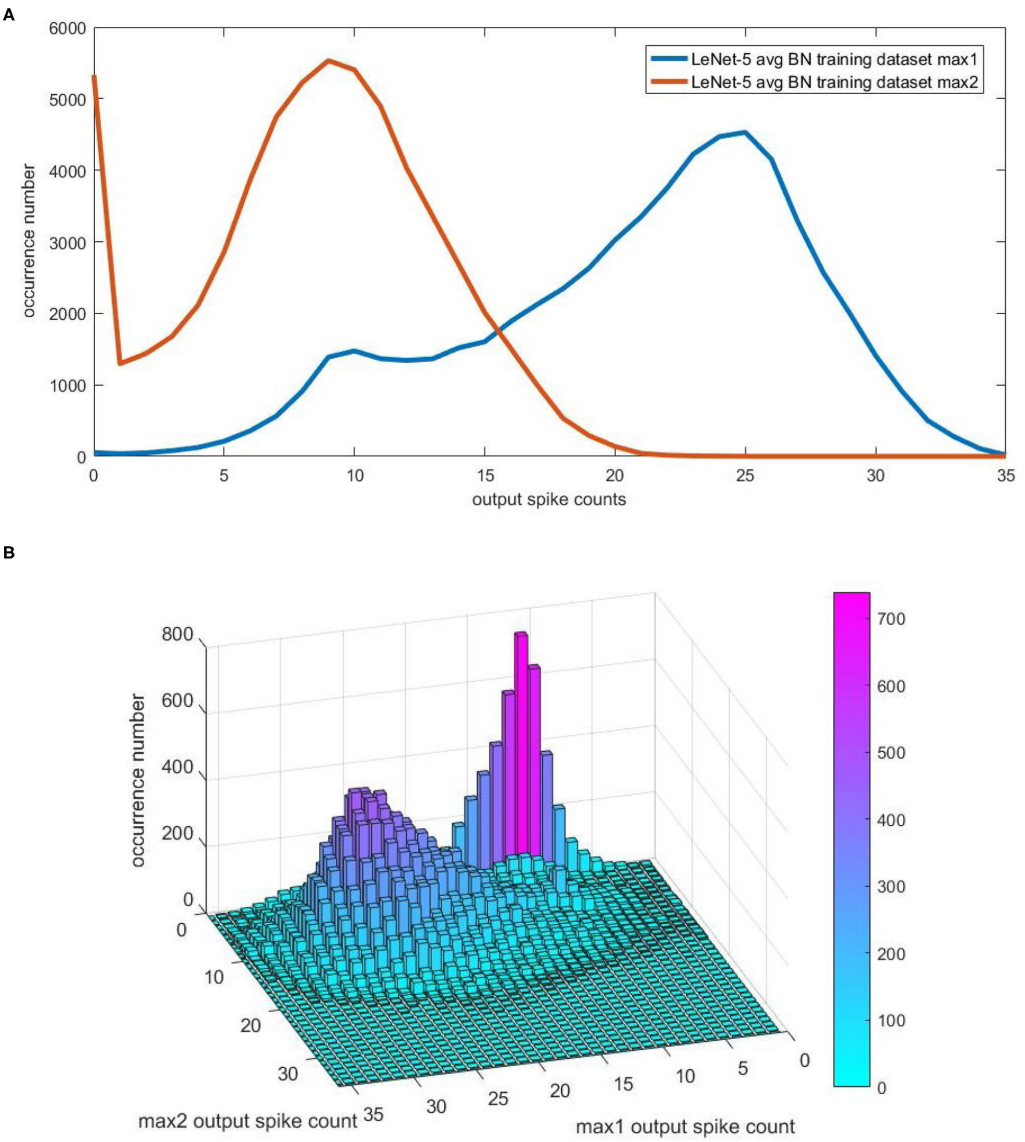
The occurrence number distribution for the output spike counts of max1 and max2 in CNN. For convenience, we use max1 and max2 to represent maximal value and second maximal value among neurons in the last layer of CNN. For Spiking-LeNet-5(avg, BN, analog) and training dataset, occurrence numbers of different output spike counts corresponding to max1 and max2 at different time steps have been recorded. **(A)** The occurrence number distribution after 40 time steps, counting, respectively, for max1 and max2. **(B)** The occurrence number distribution after 40 time steps, counting simultaneously for max1 and max2.

For convenience, we use max1 and max2 to refer to the maximal value and second maximal value among neurons in the last layer of CNN. For Spiking-LeNet-5 (avg, BN, analog) model, we record all the output spike counts corresponding to max1 and max2 after going through training dataset. For different output spike counts, we count their occurrence numbers at different time steps. Figure 4A exhibits the occurrence number distribution after 40 time steps, counting, respectively, for max1 and max2. As can be seen from this figure, there is a distinct gap between output spike counts for max1 and max2. Figure 4B displays the occurrence number distribution after 40 time steps, counting simultaneously for max1 and max2. As we know nothing of testing dataset, what we can rely on is training dataset. These occurrence number distributions will be important research materials for models with biases.

### 2.4. Proposed Inference-Latency Reducing Plug-Ins

**Figure 5:**
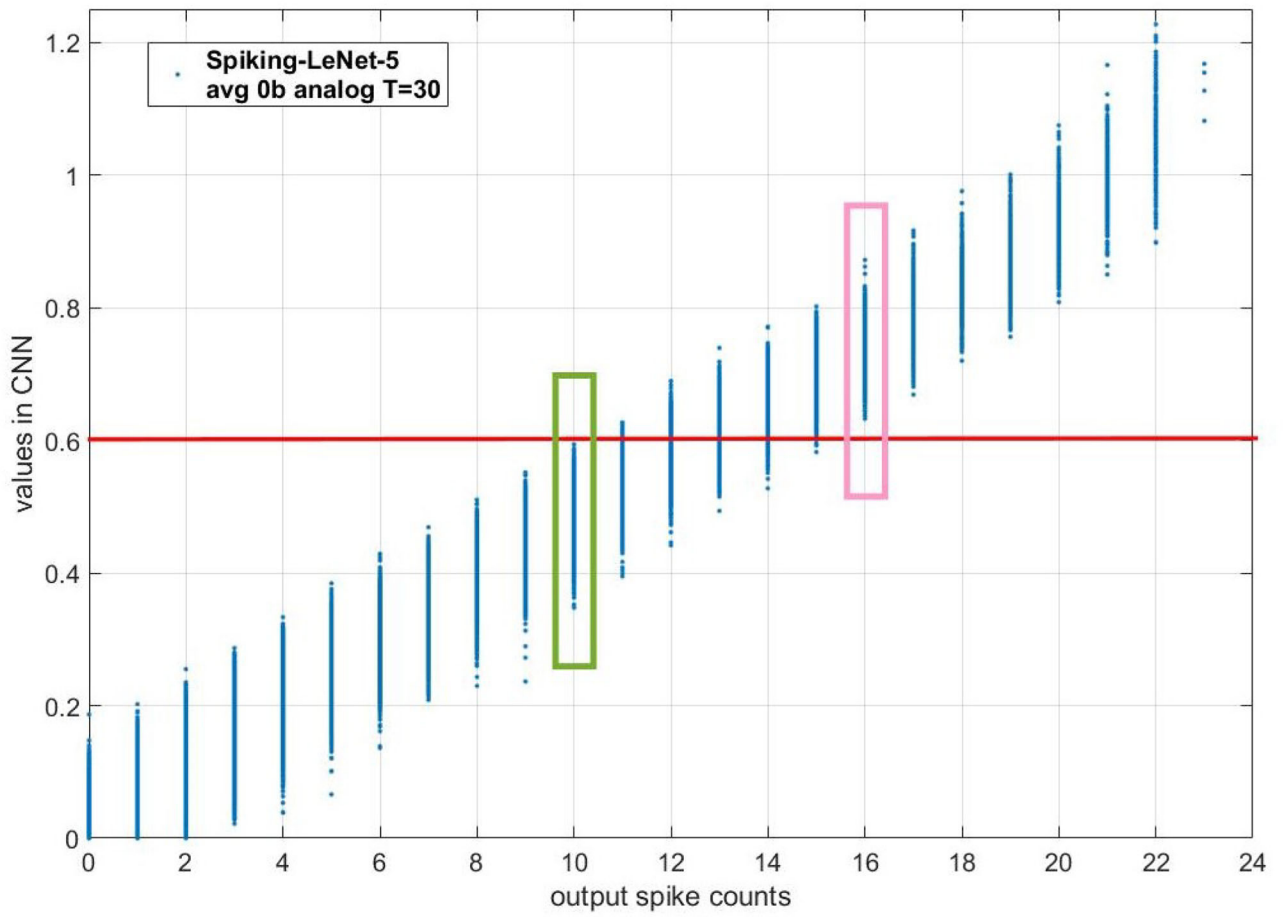
The scatter diagram of different CNN values and their corresponding output spike counts when T is 30 time steps for Spiking-LeNet-5(avg, 0b, analog) model. The red horizontal line means CNN value 0.6. The output spike count of values in green box is 10. The output spike count of values in pink box is 16. Obviously, all the values in green box are not more than 0.6 while all the values in pink box are greater than 0.6. Therefore, when we receive two output spike counts 10 and 16, we are sure that CNN value corresponding to output spike count 16 is greater than that corresponding to output spike count 10.

In section 2.3.2, we have observed that for models with no biases, there is a linear relation between the fitted mean of possible values in CNNs and output spike count in SCNNs. We can deduce that for two CNN values with a big gap, their output spike counts will own the same size relationship with high probability. To illustrate this deduction, we draw the scatter diagram of different CNN values and their corresponding output spike counts when T is 30 time steps for Spiking-LeNet-5(avg, 0b, analog) model, as is shown in Figure 5. The red horizontal line means CNN value 0.6. The output spike count of values in green box is 10. The output spike count of values in pink box is 16. Obviously, all the values in green box are not more than 0.6 while all the values in pink box are greater than 0.6. Therefore, when we receive two output spike counts 10 and 16, we are sure that CNN value corresponding to output spike count 16 is greater than that corresponding to output spike count 10. By analogy, when we receive two output spike counts with a big gap, their CNN values will own the same size relationship. According to this deduction, section 2.4.1 proposes a simple stopping criterion with enumeration, section 2.4.2 puts forward a stopping criterion based on Normal distribution theory and section 2.4.3 presents a simple stopping criterion based on Normal distribution theory. For situation with biases, section 2.4.4 brings forward a stopping criterion based on the estimation of training dataset in section 2.3.3.

#### 2.4.1. Simple Stopping Criterion With Enumeration

No matter 0b or BN models, to differentiate output spike counts corresponding to different values, the simplest solution is subtraction. In this way, we set a simple stopping criterion for SCNNs:


(4) $$\begin{array}{l} Count1=max(output\;spike\;counts(T))\\ Count2=max(output\;spike\;counts(T)\;without\;Count1)\\ If\;Count1\;\geq \;Count2+REQ\;or\;T=T_{max},\;end\\ else\;T=T+1,\;continue. \end{array}$$

In this stopping criterion, we use REQ to represent the gap between Count1 and Count2, which means the requirement for this gap. When the stopping criterion of REQ is satisfied, we regard the class of Count1 as our determination. For tough data, which will not easily meet the stopping criterion of REQ, we use T_*max*_ to limit their running time, avoiding meaningless waste of time. For other data, Count1 is larger than Count2 at time steps before T_*max*_. With this stopping criterion, these data will certainly save inference-latency. We can use the same T_*max*_ as original models but need to find suitable REQ by enumeration and multiple experiments.

#### 2.4.2. Stopping Criterion Based on Normal Distribution Theory

According to the observation in section 2.3.2, for models without biases, we can differentiate output spike counts by Normal distribution theory. After T time steps, value *X*_1_ in CNNs, which corresponds to output spike count Count1 in SCNNs, is normally distributed. The mean of this normal distribution is defined as μ_1_(*T*) and the standard deviation is defined as σ_1_(*T*). Similarly, value *X*_2_ in CNNs, which corresponds to output spike count Count2 in SCNNs, obeys normal distribution. The mean is μ_2_(*T*) and the standard deviation is σ_2_(*T*). We assume that Count1 is larger than Count2 and define X equals to *X*_1_ minus *X*_2_. We can deduce following inferences from normal distribution theory:


(5) $$\begin{array}{l} X_{1}\sim \text{N}\left(\mu_{1}\left(T\right),\sigma_{1}{\left(T\right)}^{2}\right)\;while\;Count1\\ X_{2}\sim \text{N}\left(\mu_{2}\left(T\right),\sigma_{2}{\left(T\right)}^{2}\right)\;while\;Count2\\ X=X_{1}-X_{2},Count2<Count1\\ \text{X}\sim \text{N}\left(\mu_{1}\left(T\right)-\mu_{2}\left(T\right),\sigma_{1}{\left(T\right)}^{2}+\sigma_{2}{\left(T\right)}^{2}\right)\\ \text{P}\left(X_{1}<X_{2}\right)=\text{P}\left(\text{X}<0\right). \end{array}$$

In this way, μ(*T*) and σ(*T*) fitted in section 2.3.2 can be used to calculate the degree of credibility for each combination of Count1 and Count2. As there are errors existing from fitting process and different datasets, the probability calculated by Normal distribution theory is not actual probability for testing dataset. The value can only represent an extent for reference. We define P(X<0) as an extent of error (EE). With set EE, stopping criterion based on Normal distribution theory is as follows:


(6) $$\begin{array}{l} \begin{array}{c} Count1=max\left(output\;spike\;counts\left(T\right)\right)\\ Count2=max\left(output\;spike\;counts\left(T\right)\;without\;Count1\right)\\ X\sim \text{N}\left(\mu_{1}\left(T\right)-\mu_{2}\left(T\right),\sigma_{1}{\left(T\right)}^{2}+\sigma_{2}{\left(T\right)}^{2}\right)\\ IfP\left(X<0\right)<EE\;or\;T=T_{max},end\\ else\;T=T+1,continue. \end{array} \end{array}$$

#### 2.4.3. Simple Stopping Criterion Based on Normal Distribution Theory

In section 2.3.2, we have observed that μ(*T*) is nearly linear and σ(*T*) is approximately horizontally linear for models without biases. Therefore, we put the slope of μ(*T*) and the mean value of σ(*T*) into our equation in section 2.4.2 and name them k(T) and $\bar{\sigma }$(T), respectively:


(7) $$\begin{array}{l} \begin{array}{c} Count_{1}-Count_{2}=REQ\\ \mu_{1}\left(T\right)-\mu_{2}\left(T\right)\approx k\left(T\right)*\left(Count_{1}-Count_{2}\right)=k\left(T\right)*REQ\\ \sigma_{1}{\left(T\right)}^{2}+\sigma_{2}{\left(T\right)}^{2}\approx {\left[\sqrt{2}\bar{\sigma }\left(T\right)\right]}^{2}\\ P\left(X<0\right)<EE,\text{X}\sim \text{N}\left(k\left(T\right)*REQ,{\left[\sqrt{2}\bar{\sigma }\left(T\right)\right]}^{2}\right)\\ \text{P}\left(\text{Y}<-\frac{k\left(T\right)*REQ}{\sqrt{2}\bar{\sigma }\left(T\right)}\right)<EE,\text{Y}\sim \text{N}\left(0,1\right)\\ REQ>\frac{\sqrt{2}\bar{\sigma }\left(T\right)*norminv\left(1-EE,0,1\right)}{k\left(T\right)}. \end{array} \end{array}$$

Norminv function means inverse cumulative distribution function (ICDF) of normal distribution. For actual use, REQ should round up into an integer by ceil function. Compared with simple stopping criterion with enumeration, REQ used in this section is calculated by set EE and fitted Normal distribution parameters:


(8) $$\begin{array}{l} Count1=max(output\;spike\;counts(T))\\ Count2=max(output\;spike\;counts(T)\;without\;Count1)\\ REQ(EE,T)=\frac{\sqrt{2}\bar{\sigma }(T)*norminv(1-EE,0,1)}{k(T)}\\ If\;Count1\;\geq \;Count2+ceil\left(REQ(EE,T)\right)\;or\;T=T_{max},\;end\\ else\;T=T+1,\;continue. \end{array}$$

**Figure 6:**
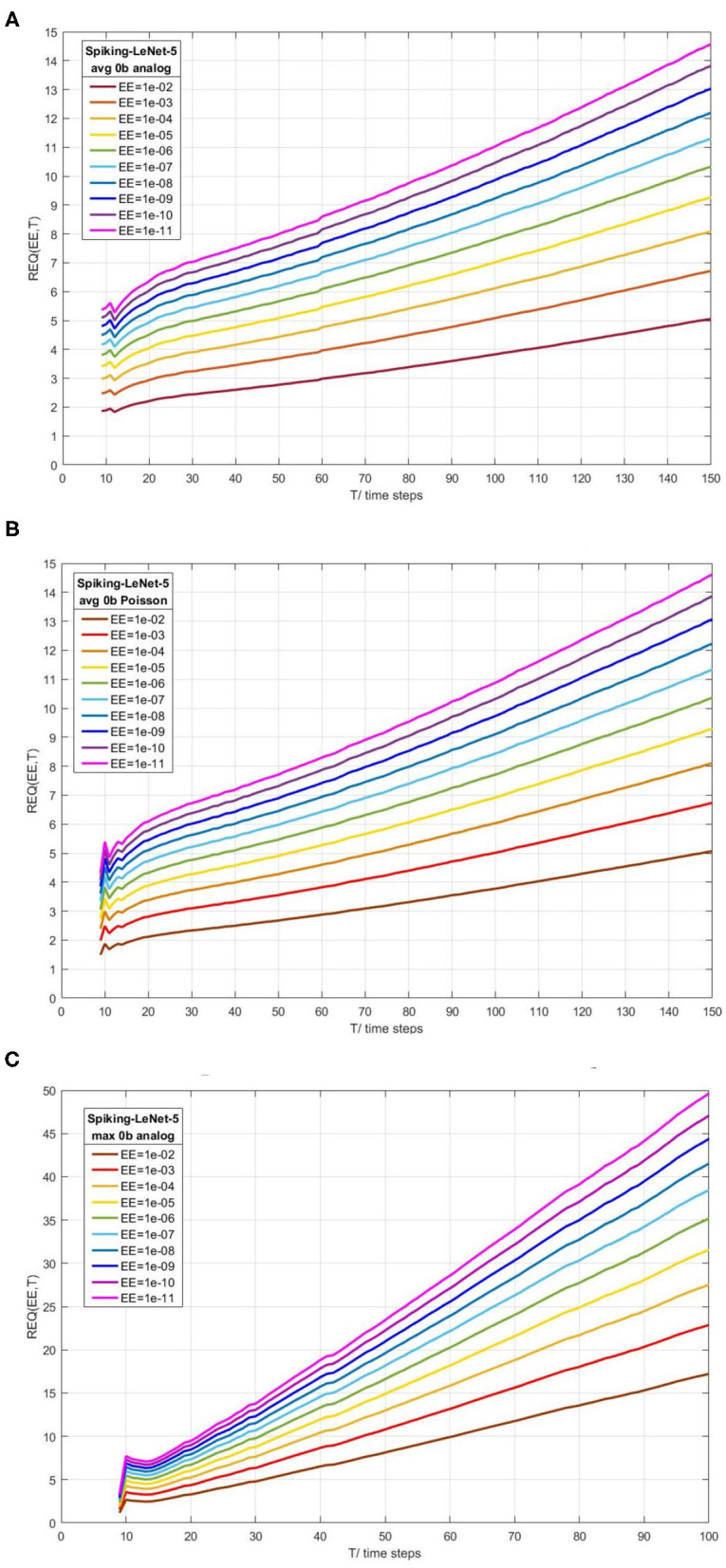
Calculated REQ(EE,T) according to Normal distribution theory for Spiking-LeNet-5(avg, 0b, analog), Spiking-LeNet-5(avg, 0b, Poisson) and Spiking-LeNet-5(max, 0b, analog), respectively. EE means extent of error. T means time steps. REQ is proposed in simple stopping criterion with enumeration. **(A)** Spiking-LeNet-5 avg 0b analog. **(B)** Spiking-LeNet-5 avg 0b Poisson. **(C)** Spiking-LeNet-5 max 0b analog.

Figures 6A–C exhibit REQ(EE,T) for Spiking-LeNet-5 (avg, 0b, analog), Spiking-LeNet-5 (avg, 0b, Poisson) and Spiking-LeNet-5 (max, 0b, analog), respectively. Figures 6A,B are similar as they share the same weights and network architectures, except for REQ(EE,T) before 15 time steps. This difference is caused by different input forms. The slopes of REQ(EE,T)-T in Figure 6C are visibly larger than others, which indicates that Spiking-LeNet-5(max, 0b, analog) needs larger REQ than other models to get similar performance. This phenomenon comes from the larger standard deviations of Max-pooling models as mentioned in section 2.3.2. From the three figures, we can see that smaller EE and larger T will lead to larger REQ. In the cases of fixed EE, REQ is nearly linear to T. Performance of this stopping criterion can help us choose suitable REQ for stopping criterion in section 2.4.1.

#### 2.4.4. Stopping Criterion Based on the Estimation of Training Dataset

From section 2.3.1, we have found that, for models with biases, it's hard to describe the relation between CNN values and output spike counts through directly fitting the distributions. Though stopping criterion with enumeration proposed in section 2.4.1 can be used in this situation, we still need to raise this problem and try to solve it.

In section 2.3.3, we have calculated occurrence number distribution of max1 output spike count (output spike count of max1 value in CNN) and max2 output spike count (output spike count of max2 value in CNN) at different time steps, denoting as ON(Count1,Count2,T). With this occurrence number distribution, when we use REQ as our stopping criterion, we can approximatively calculate the error probability P_*error*_(REQ,T) as follows:


(9) $$\begin{array}{l} P_{error}\left(REQ,T\right)\\ =\frac{\sum_{Count1-Count2\geq REQ}ON\left(Count2,Count1,T\right)}{\sum_{Count1-Count2\geq REQ}\left(ON\left(Count1,Count2,T\right)+ON\left(Count2,Count1,T\right)\right)}. \end{array}$$

The stopping criterion based on the estimation of training dataset is described as follows:


(10) $$\begin{array}{l} \begin{array}{c} Count1=max\left(output\;spike\;counts\left(T\right)\right)\\ Count2=max\left(output\;spike\;counts(T)\;without\;Count1\right)\\ IfP_{error}\left(Count1-Count2,T\right)<EE\;or\;T=T_{max},end\\ else\;T=T+1,continue. \end{array} \end{array}$$

We still use EE to represent the extent of error. The core of this stopping criterion is to estimate the size relationship of output spike counts for testing dataset by training dataset through calculated error probabilities.

## 3. Results

Except for the training of CNNs, all the experiments and figures in this section are done on MATLAB platform. We get all the distribution data from training dataset. The testing results in this section are due to testing dataset. For AlexNet, we use CIFAR-10 dataset. For LeNet-5, we use MNIST dataset.

### 3.1. Performance of Proposed Methods

For convenience, we use stopping criterion 1 to denote the simple stopping criterion with enumeration in section 2.4.1, stopping criterion 2 to denote the stopping criterion based on Normal distribution theory in section 2.4.2, stopping criterion 3 to denote the simple stopping criterion based on Normal distribution theory in section 2.4.3 and stopping criterion 4 to denote the stopping criterion based on the estimation of training dataset in section 2.4.4.

We firstly select suitable T_*max*_ for each original model to get suitable accuracy. In our proposed stopping criterions, we use the same T_*max*_ as that in original models. During inference phase, we sum all the inference-latency for testing dataset and calculate the average inference-latency. To exhibit the performance of proposed stopping criterions on the reduction of inference-latency, we use the ratio of average inference-latency to T_*max*_. We also compare the accuracies when using proposed stopping criterions or original one, denoting their difference as accuracy decline.

#### 3.1.1. Performance of Stopping Criterion 1

The accuracies of Spiking-LeNet-5(avg, 0b, analog) are 98.50, 98.51, and 98.56% when REQ is 5, 6 and 7, respectively. Their average inference-latencies are 24, 27, and 30 time steps, while T_*max*_ is 56 and the accuracy of original model is 98.59%. For Spiking-LeNet-5(avg, 0b, Poisson), accuracies of 98.48, 98.50, and 98.52% can be achieved with the REQ of 6, 7, and 8, while the accuracy of original model is 98.57% at 52 time steps. Corresponding inference-latencies are 27, 30, 33 time steps. The original model of Spiking-LeNet-5(max, 0b, analog) obtains the accuracy of 97.99% at 96 time steps. With the REQ of 6, 8, and 10, accuracies of 97.91, 97.94, and 98.00% can be achieved at 30, 37, 43 time steps on average. For Spiking-LeNet-5(avg, BN, analog), accuracies of 98.72, 98.77, and 98.80% can be reached with the REQ of 15, 18, and 20 when inference-latencies are 70, 76, and 80, respectively. The accuracy of original model is 98.82% at 138 time steps. Original Spiking-AlexNet(avg, 0b, analog) model can achieve the accuracy of 87.95% at 892 time steps. With the REQ of 25, 27, and 29, the accuracies of 87.65, 87.67, and 87.72% can be reached at 243, 255, and 267 time steps.

**Figure 7:**
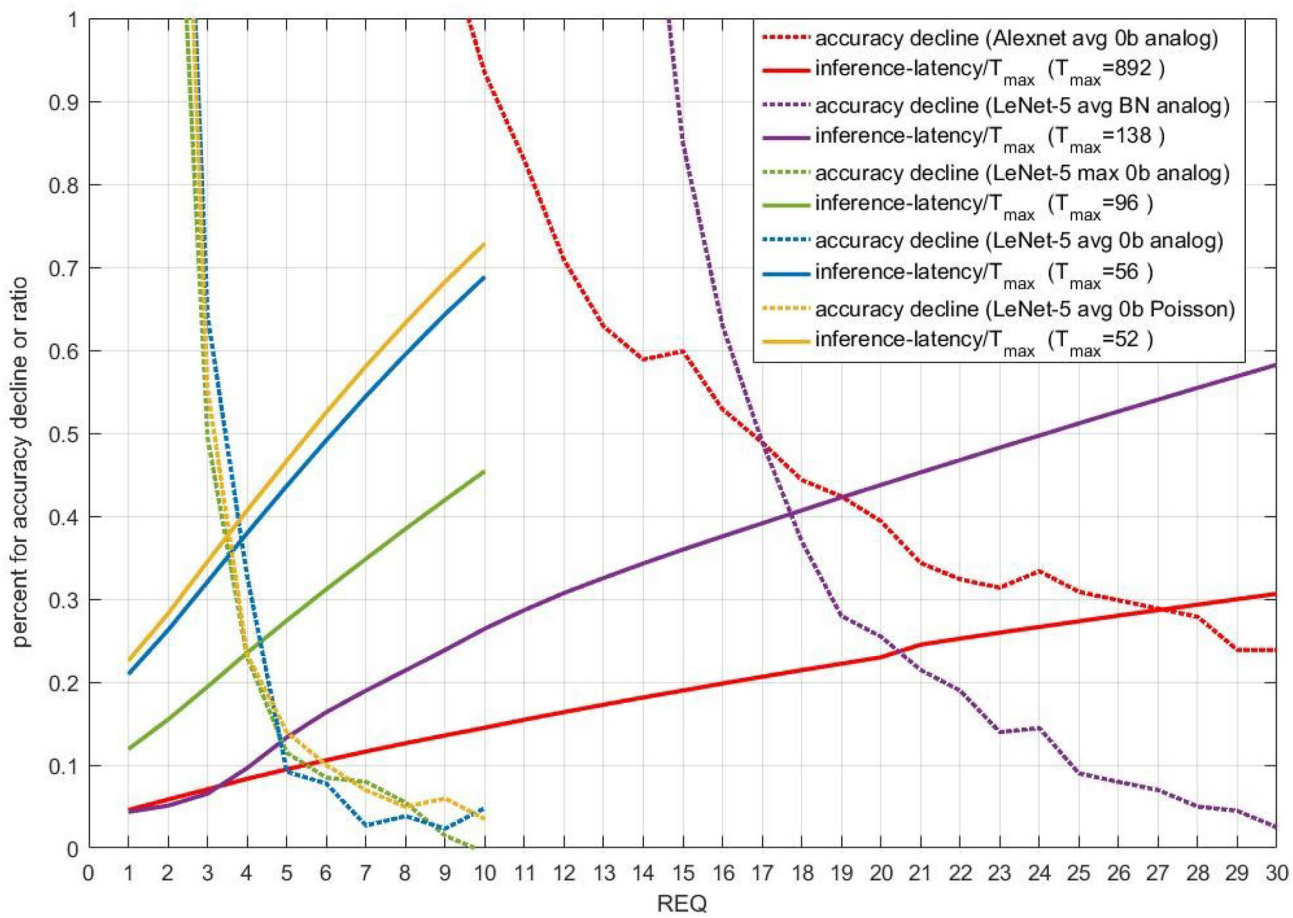
Performance of stopping criterion 1 for five different models. X-axis is REQ. For full lines, Y-axis means ratio of average inference-latency to T_*max*_. For dotted lines, Y-axis means accuracy decline compared with original models owning the same T_*max*_, the unit of which is percent. Different colors means different models.

Performance of stopping criterion 1 for five different models is displayed in Figure 7. The dotted lines are accuracy decline. LeNet-5 without biases can achieve good accuracy performance with the REQ smaller than 10. For AlexNet and LeNet-5 with biases, the REQ near 30 is suitable. When REQ is large enough, the accuracy improvement is unapparent compared with smaller REQ. We can deduce that accuracy can be improved with the increase of REQ in one certain range. What's more, the average inference-latency is nearly linear to REQ for most models. When accuracy decline is smaller than 1%, the ratio of average inference-latency to T_*max*_ is in the range of 0.1 to 0.7. The tradeoff between accuracy and inference-latency is evident in Figure 7. By enumeration, users can select the smallest REQ to get the best accelerating performance, while satisfying their accuracy demands.

#### 3.1.2. Performance of Stopping Criterion 2 and 3

For stopping criterion 2, the accuracies of Spiking-LeNet-5(avg, 0b, analog) are 98.50, 98.52, and 98.56% when EE is 10e-6, 10e-8, and 10e-10, respectively. Their average inference-latencies are 24, 27, and 30 time steps, while T_*max*_ is 56 and the accuracy of original model is 98.59%. With the same EE, for stopping criterion 3, the accuracies of Spiking-LeNet-5(avg, 0b, analog) are 98.49, 98.52, and 98.53% consuming 25, 28 and 31 time steps. For Spiking-LeNet-5(avg, 0b, Poisson), the accuracies of stopping criterion 2 with the EE of 10e-6, 10e-8, and 10e-10 are 98.44, 98.48, and 98.51%, while corresponding inference-latencies are 24, 27, and 29 time steps. With stopping criterion 3 and the same EE, the accuracies for Poisson model are 98.41, 98.49, and 98.51% at 24, 27, and 29 time steps, respectively. The accuracy of original Poisson model is 98.57% at 52 time steps. In the case of Spiking-LeNet-5(max, 0b, analog), using stopping criterion 2 can achieve the accuracies of 97.68, 97.90, and 97.99% with the EE of 10e-3, 10e-4, and 10e-5, while corresponding inference-latencies are 33, 44, and 56 time steps. With stopping criterion 3 and the same EE, accuracies of 97.92, 97.99, and 97.98% can be reached at 40, 54, and 64 time steps. For original model, the accuracy is 97.99% at 96 time steps.

**Figure 8:**
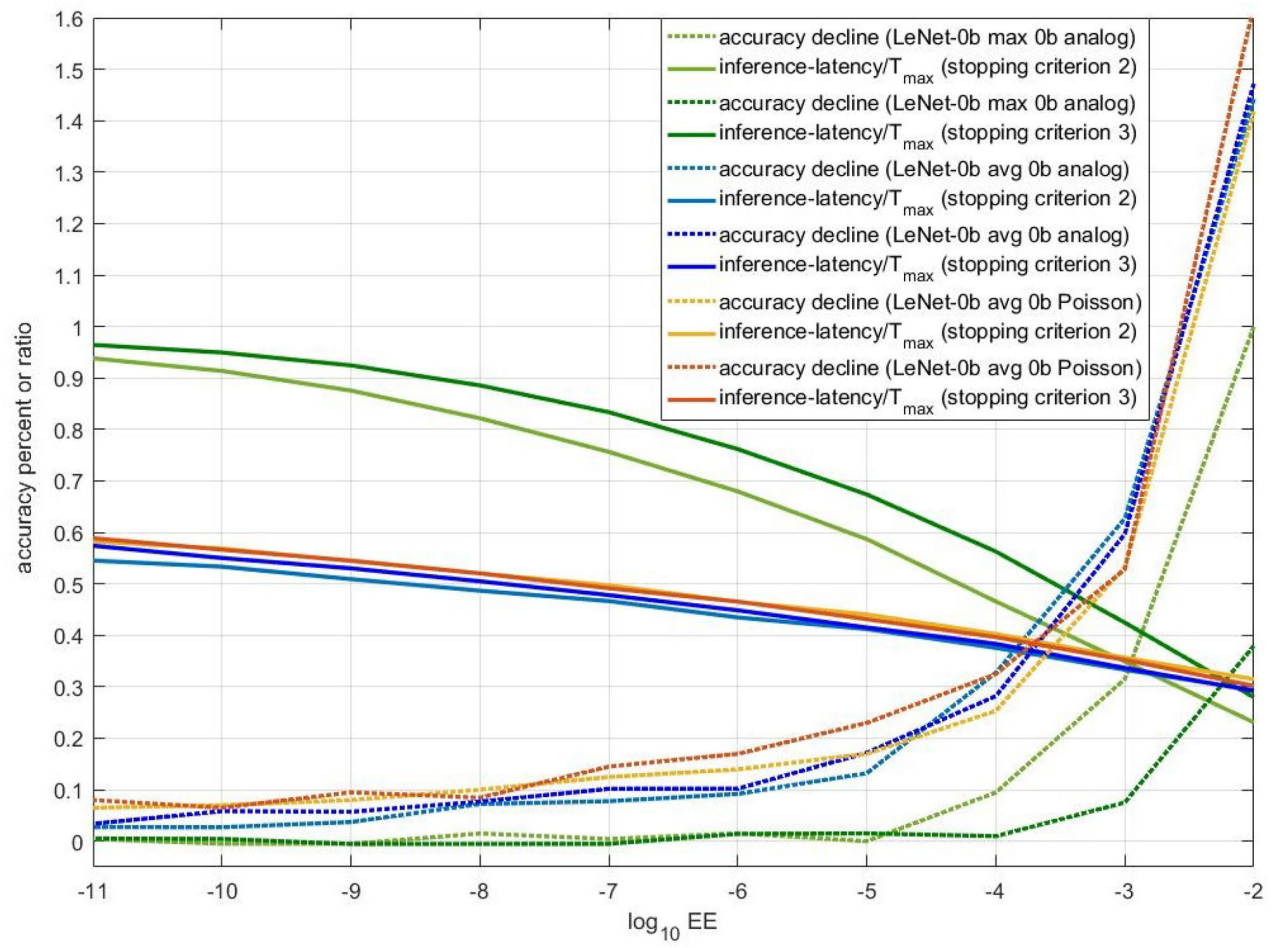
Performance of stopping criterion 2 and 3 for three different models. X-axis is REQ. For full lines, Y-axis means ratio of average inference-latency to T_*max*_. For dotted lines, Y-axis means accuracy decline. The unit is percent.

As stopping criterion 2 and stopping criterion 3 share the same principle and parameter EE, we compare their performance in Figure 8. In this figure, when EE is smaller than 1e-02, accuracy decline is not more than 1.6%. For Avg-pooling models, stopping criterion 2 and 3 achieve similar ratio of average inference-latency to T_*max*_ (reciprocal of accelerative ratio) and accuracy decline. With the accuracy decline near 0.3%, the accelerative ratio is close to 2.5X. For Max-pooling models, when EE is smaller than 1e-05, stopping criterion 2 and 3 share similar accuracy decline. The accelerating performance of stopping 3 is a little bit worse than that of stopping criterion 2. With the accuracy decline near 0.3%, the accelerative ratio is also close to 2.5X. For actual use, if computing resources are limited, replacing complex lookup table of fitted means and standard deviations (stopping criterion 2) by several simple REQs (stopping criterion 3) will be better.

**Figure 9:**
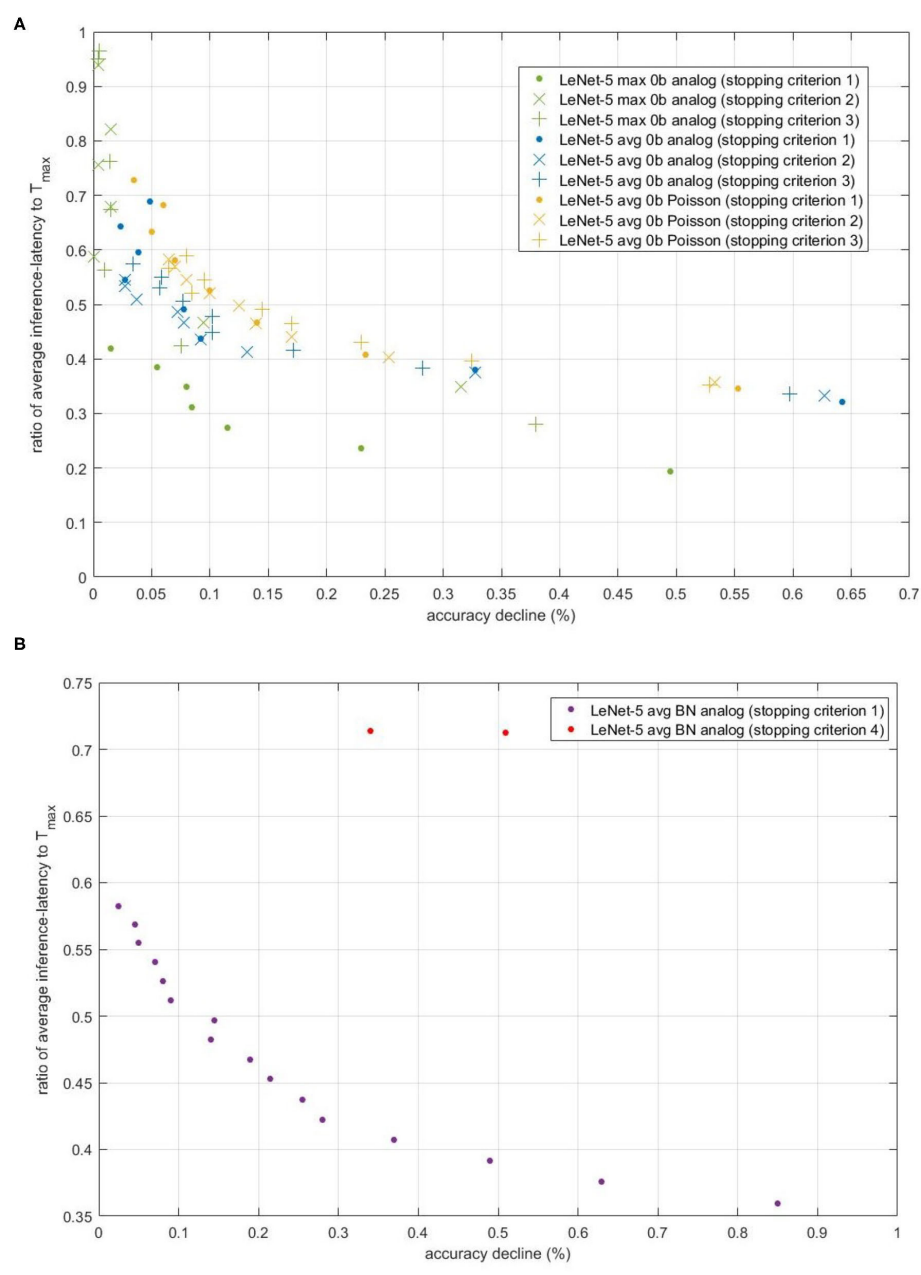
The scatter of (accuracy decline, ratio of average inference-latency to T_*max*_) for different models and different stopping criterions. The x-axis is accuracy decline, unit of which is percent. The y-axis is the ratio. **(A)** Comparison of the accuracy-inference-latency tradeoff among stopping criterion 1, 2, and 3 for three models. **(B)** Comparison of the accuracy-inference-latency tradeoff between stopping criterion 1 and 4 for Spiking-LeNet-5 avg BN analog model.

Figure 9A compares the accuracy-inference-latency tradeoff among stopping criterion 1,2 and 3. Taking both accuracy and inference-latency into consideration, stopping criterion 1 for LeNet-5 Max-pooling model has the best performance. Though the accelerating performance of stopping criterion 2 and 3 for LeNet-5 Max-pooling model is a little bit worse than that of stopping criterion 1, the accuracy decline of stopping criterion 2 and 3 can be ensured. For avg-0b models, performance of the three stopping criterions is similar. The performance of analog model is better than Poisson model. According to this figure, for avg-0b models, we can use stopping criterion 3 to calculate suitable REQ beforehand, avoiding the enumeration of REQ in stopping criterion 1. In fact, based on Figure 9A, when EE is smaller than 1e-08, accuracy decline will be stable. Through formula introduced in section 2.4.3, the REQ can be calculated with this set EE.

#### 3.1.3. Performance of Stopping Criterion 4

Figure 9B compares the accuracy-inference-latency tradeoff between stopping criterion 1 and 4 for LeNet-5 BN model. Stopping criterion 4 seems terrible as its ratio is near 0.7 with accuracy decline more than 0.3%. For actual use, to deal with models with biases, stopping criterion 1 may be better choice. The terrible performance of stopping criterion 4 may be caused by the gap between training set and testing set. Compared with stopping criterion 2 and 3, stopping criterion 4 only uses 60,000 numbers to calculate occurrence number distribution, while others use nearly 300,000 numbers (assuming that half of them are negative) to make normal fitting and then extract rules and information from fitting results.

### 3.2. Comparison With Others

Table 1 compares inference latency and accelerative ratio between our proposed method and other works. Neil et al. (2016) introduced several optimization approaches during training phase to reduce the inference-latency of SCNNs. However, to achieve the accuracy of 98.00% for MNIST, all of these approaches need more than 600 time steps. By contrast, our models only need 24–70 time steps.

**Table 1:** Comparison with other inference-latency reducing methods.

**Dataset**	**Method**	**Accuracy (decline compared with CNN)**	**Inference-latency (time steps)**	**Accelerative ratio**
MNIST (Neil et al., 2016)	Sparse Coding	98.00%	631	-
MNIST (Neil et al., 2016)	Activation Cost	98.00%	602	-
MNIST (Neil et al., 2016)	Dropout	98.00%	641	-
MNIST (Neil et al., 2016)	Dropout Learning Sched.	98.00%	602	-
MNIST (Neil et al., 2016)	Stacked AE	98.00%	788	-
MNIST (Avg 0b Analog)	Stopping criterion	98.50% (0.06%)	24	1.88X
MNIST (Avg 0b Poisson)	Stopping criterion	98.48% (0.08%)	27	1.48X
MNIST (Max 0b Analog)	Stopping criterion	97.91% (0.74%)	30	1.97X
MNIST (Avg BN Analog)	Stopping criterion	98.73% (0.09%)	70	1.39X
MNIST (Yang et al., 2020)	Conversion rule	99.03% (0.08%)	67	1.49X
CIFAR-10 (Avg 0b Analog)	Stopping criterion	87.72% (0.23%)	267	1.87X
CIFAR-10 (Avg 0b Analog)	Stopping criterion	87.25% (0.70%)	146	1.81X
CIFAR-10 (Yang et al., 2020)	Conversion rule	80.03% (0.78%)	245	1.63X

*The accelerative ratio in the table is compared with original model (Rueckauer et al., 2017) under the same accuracy*.

Our experiments add proposed stopping criterions on original models, which use the same neuron equations, the same threshold generating principle and the same principle of biases and Max-pooling layers as the work of Rueckauer et al. (2017). The accelerative ratio in Table 1 is the ratio of the average inference-latency of original models to the average inference-latency of new models under the same accuracy. From the table we can see that the accelerative ratios of our methods are in the range of 1.39X to 1.97X without accuracy loss. The work of Yang et al. (2020) is also an improvement of the work of Rueckauer et al. (2017). In their work, deterministic conversion rule for CNNs to SCNNs is proposed to reduce inference-latency, mainly concentrating on weights and thresholds. Because of different basic trained CNNs, we compare the accuracy decline between SCNNs and CNNs. For CIFAR-10 dataset, they achieve the accuracy of 80.03% and the accuracy decline is 0.78%. We find the data point with similar accuracy decline 0.70%. Our model needs 146 time steps and their model needs 245 time steps. Considering the accelerative ratio which is compared with the work of Rueckauer et al. (2017), our work performs better.

Besides, these methods mainly concentrate on training phase, weights, and thresholds, our methods mainly concentrate on the stopping criterion of inference phase. As plug-ins, our stopping criterions have been demonstrated effective on the basis of the work of Rueckauer et al. (2017), which have improved the inference-latency by weight normalization. Therefore, there is no theoretical conflict between our stopping criterions and other inference-latency reducing methods such as adjusting thresholds or weight normalization. So long as there is a gap between maximal and the second maximal output spike counts, it is possible to combine our plug-ins with these methods to get better performance.

## 4. Discussion

Converting from CNNs, SCNNs have the advantage of accuracy compared with SNNs. By using spikes as data flows, SCNNs are much more energy-saving than CNNs. However, inference-latency, i.e., the processing speed of classification, cannot be ignored in practical SCNN applications (Roy et al., 2019). Thus, the stopping criterions proposed in this work aim to reduce the inference-latency of SCNNs. Experiment results have demonstrated that our stopping criterions can be used in SCNNs as plug-ins for several different network components. With few extra computing resources, our plug-ins can significantly speed up the inference phase of SCNNs in hardware implementations.

In section 2.1, our original models are built on the basis of the work of Rueckauer et al. (2017), which represents the state-of-the-art and complete theory of SCNNs, including techniques such as the implementation of Max-pooling layers and batch normalization layers, and the normalization of weights and biases for each layers. For original models, after observing the relation between accuracy and inference-latency, we can draw the conclusion that accuracy increases fast with the increment of inference-latency at first, then increases slowly and finally becomes stable near a fixed value. Simply reducing inference-latency will lead to the loss of accuracy. Focusing on incorrectly classified data, we find some of them have one common feature, that in the last layer, the maximal counter and the second maximal counter will receive similar number of spikes. The determination for these cases is hard to make and time-consuming. We define these data as tough data. Through analyzing the gap among output spike counts of each data point in training dataset, we can deduce some rules to distinguish tough data and other data. Allocating more time steps for tough data and fewer time steps for other data can reduce total inference-latency and ensure accuracy simultaneously.

### 4.1. Connection With Cognitive Neuroscience

In section 2.2, we find tough data hard to obtain correct results even after consuming a considerable amount of time steps. On the contrary, other data can achieve the same results as CNNs without too many time steps. During inference phase, we need to make the termination decision if current output spike counts meet our preset requirement. According to our analysis of output spike counts, we use the gap between the largest output spike count and the second largest output spike count as the key index in stopping criterion 1. Experiment results reveal that stopping criterion 1 can achieve good performance. Moreover, higher requirement of the gap (REQ) will lead to higher accuracy but larger inference-latency. In psychology, similar balance when people and animals make decisions under time pressure is known as the speed-accuracy tradeoff (Latty and Beekman, 2011). Sequential sampling models assume that decision maker accumulates noisy samples of information from the environment until a threshold of evidence is reached (Ratcliff and Smith, 2004; Bogacz et al., 2006; Teodorescu and Usher, 2013; Forstmann et al., 2015). REQ in stopping criterion 1 has similar effect as the threshold of evidence in sequential sampling models, demonstrating that our stopping criterion for SCNNs is conformed to the cognitive law of organic brains.

Except making difference directly, in stopping criterion 2 and 3, we also use Normal distribution theory to calculate the error probability according to current output spike counts. In stopping criterion 4, we use the occurrence number distribution in training dataset to estimate the error probability in testing dataset. In visual tasks, Visual confidence(VC) refers to the ability of the observer to make a good inference on the validity of the response corresponding to this perceptual decision (Mamassian, 2016). Though VC is used in training phase rather than inference phase, the usage of probability in our stopping criterions is similar to VC, providing reasonability for our proposed error probability.

Nevertheless, terminal condition in programming algorithm is different from that in brain. In our stopping criterions, we use a fixed T_*max*_ to limit the longest inference time. In our daily life, when we make decisions, we can adjust the determining time for many reasons. For example, external disturbance, mood, and physical state can influence our decision making phase. But in our codes, we need to set a terminal condition. If output spike counts cannot fit the need of set stopping criterion after a long time, the program will stop the current inference and begin a next turn.

### 4.2. Differences Between 4 Stopping Criterions

In section 2.4, we propose 4 stopping criterions to reduce the inference-latency of SCNNs for different situations. In stopping criterion 1, we use REQ as a threshold of the difference of maximal output spike count and the second maximal output spike count. If the difference exceeds REQ, we will select the maximal counter as our final choice and stop this turn of inference. REQ here needs enumeration. In stopping criterion 2, we need to calculate the error probability of current decision in real time with two maximal output spike counts and pre-computed parameters. EE is the requirement of error probability. As the fitted means are linear and fitted standard deviations are horizontally linear to time steps in section 2.3.2, after formula derivation in section 2.4.3, we can convert the error probability into the form of REQ before inference starts. In stopping criterion 3, we use a look-up table of REQ changing with time steps as the requirement of current output spike counts, avoiding continuously calculating error probability. In stopping criterion 4, as we cannot use Normal distribution theory to deal with BN models, we propose another expression of error probability. For each data point in training dataset, we find maximal and the second maximal CNN value in the last layer and record their corresponding output spike counts in SCNNs. Error probability in this stopping criterion is calculated directly by statistical information above. Therefore, the degree of similarity between training dataset and testing dataset will affect the reliability of this stopping criterion. For all the 4 stopping criterions, we use the same T_*max*_ as original stopping criterion, limiting the meaningless inference of tough data.

For compatibility, we try 5 different models, including CIFAR-10 and MNIST, AlexNet, and LeNet-5, Avg-pooling layer and Max-pooling layer, biases set to zero and BN layer with biases, analog input and Poisson input. Stopping criterion 1 performs well for all the 5 different models. Accelerative ratio of inference-latency is in the range of 1.92X to 3.34X. For LeNet-5 models, the accuracy decline is not more than 0.1%. For AlexNet models, the accuracy decline is 0.23%. Stopping criterion 2 is useful for LeNet-5 avg models without biases. Stopping criterion 3 share similar performance as stopping criterion 2. Stopping criterion 4 cannot work as well as stopping criterion 1 for models with biases, remaining problem to solve in the future.

For actual use, we need to choose different stopping criterions for different models. For Avg-pooling models without biases, we can use stopping criterion 2 and 3. If real-time computing resources are limited, stopping criterion 3 is better. For Max-pooling models without biases, we can first use stopping criterion 3 to help us find suitable REQ range and use stopping criterion 1 later on. For models with biases, only stopping criterion 1 can be used but we can first calculate the error probability in stopping criterion 4 to find the rough range of REQ, avoiding large amount of enumeration.

### 4.3. New Problem and Possible Solutions

As bias is an important ingredient for CNNs, the inference-latency reducing method for SCNNs with biases is of great significance. According to our experiment results, stopping criterion 1 achieves better performance than stopping criterion 4. This result suggests that calculating the error probability of testing dataset through simply imitating training dataset is not enough. In fact, the difference between bias situation and 0-bias situation is the summing way of biases. In SCNNs, biases are continuously accumulated into neurons all the time but weights are accumulated into neurons only when there are input spikes. Over time, weights and biases accumulated in neurons conform to the same relationship as that in CNNs. However, at the early stage of SCNNs, disordered spike patterns are mainly contributed by biases, especially by large biases. Without changing the implementation of biases, what we can do is extracting more useful information from training dataset or adjusting the CNN values during training phase. For example, monte carlo method can be used to describe the disordered distribution in Figure 2D. What's more, if we can widen the gap between max1 and max2 output CNN values during training phase, the gap between maximal and the second maximal output spike counts will be larger to distinguish.

In summary, through adding extra stopping criterions for received output spikes at the end of inference phase, without obvious accuracy loss and extra computing resources, our proposed plug-ins can significantly reduce inference-latency of SCNNs with compatibility and organic basis.

## Data Availability Statement

The datasets presented in this study can be found in online repositories. The names of the repository/repositories and accession number(s) can be found below: https://github.com/XuanChen75/SCNN.

## Author Contributions

XC developed the theory, implemented the methods, wrote the codes for experiments, analyzed the data, and drafted the manuscript. XY trained the CNN networks and ran MATLAB simulation on CPU server. TY provided probable analysis directions. YW, YL, TY, and HP modified the structure of the manuscript. YL, GF, YW, TY, FY, and HP contributed to the writing of the manuscript. All authors contributed to the article and approved the submitted version.

## Funding

This work was supported by the National Nature Science Foundation of China under Grant Nos. 61376075 and 41412020201.

## Conflict of Interest

The authors declare that the research was conducted in the absence of any commercial or financial relationships that could be construed as a potential conflict of interest.

## Publisher's Note

All claims expressed in this article are solely those of the authors and do not necessarily represent those of their affiliated organizations, or those of the publisher, the editors and the reviewers. Any product that may be evaluated in this article, or claim that may be made by its manufacturer, is not guaranteed or endorsed by the publisher.
